# Defining Critical Genes During Spherule Remodeling and Endospore Development in the Fungal Pathogen, *Coccidioides posadasii*

**DOI:** 10.3389/fgene.2020.00483

**Published:** 2020-05-15

**Authors:** H. L. Mead, C. C. Roe, E. A. Higgins Keppler, M. C. Caballero Van Dyke, K. L. Laux, A.L. Funke, K. J. Miller, H. D. Bean, J. W. Sahl, B. M. Barker

**Affiliations:** ^1^Pathogen and Microbiome Institute, Northern Arizona University, Flagstaff AZ, United States; ^2^School of Life Sciences, Arizona State University, Tempe, AZ, United States; ^3^Center for Fundamental and Applied Microbiomics, The Biodesign Institute, Arizona State University, Tempe, AZ, United States; ^4^Imaging Histology Core Facility, Northern Arizona University, Flagstaff AZ, United States

**Keywords:** *Coccidioides*, Valley fever, dimorphic fungi, thermotolerance, spherules

## Abstract

*Coccidioides immitis* and *C. posadasii* are soil dwelling dimorphic fungi found in North and South America. Inhalation of aerosolized asexual conidia can result in asymptomatic, acute, or chronic respiratory infection. In the United States there are approximately 350,000 new infections per year. The *Coccidioides* genus is the only known fungal pathogen to make specialized parasitic spherules, which contain endospores that are released into the host upon spherule rupture. The molecular determinants involved in this key step of infection remain largely elusive as 49% of genes are hypothetical with unknown function. An attenuated mutant strain *C. posadasii* Δ*cts2*/Δ*ard1*/Δ*cts3* in which chitinase genes 2 and 3 were deleted was previously created for vaccine development. This strain does not complete endospore development, which prevents completion of the parasitic lifecycle. We sought to identify pathways active in the wild-type strain during spherule remodeling and endospore formation that have been affected by gene deletion in the mutant. We compared the transcriptome and volatile metabolome of the mutant Δ*cts2*/Δ*ard1*/Δ*cts3* to the wild-type C735. First, the global transcriptome was compared for both isolates using RNA sequencing. The raw reads were aligned to the reference genome using TOPHAT2 and analyzed using the Cufflinks package. Genes of interest were screened in an *in vivo* model using NanoString technology. Using solid phase microextraction (SPME) and comprehensive two-dimensional gas chromatography – time-of-flight mass spectrometry (GC × GC-TOFMS) volatile organic compounds (VOCs) were collected and analyzed. Our RNA-Seq analyses reveal approximately 280 significantly differentially regulated transcripts that are either absent or show opposite expression patterns in the mutant compared to the parent strain. This suggests that these genes are tied to networks impacted by deletion and may be critical for endospore development and/or spherule rupture in the wild-type strain. Of these genes, 14 were specific to the *Coccidioides* genus. We also found that the wild-type and mutant strains differed significantly in their production versus consumption of metabolites, with the mutant displaying increased nutrient scavenging. Overall, our results provide the first targeted list of key genes that are active during endospore formation and demonstrate that this approach can define targets for functional assays in future studies.

## Introduction

Fungal infections have become an increasing threat to human health, claiming the lives of ∼1.5 million people worldwide each year (Brown et al., 2012). Dimorphic fungi, which have evolved the ability to switch between specialized environmental or host-specific lifecycles, are a major cause of the increase in cases (Van Dyke et al., 2019). These fungi can cause disease in both immune-compromised and competent individuals, and this is attributed to their ability to shift to a host specific lifecycle at 37°C. *Coccidioides immitis* and *C. posadasii* are two of these dimorphic fungi associated with animals/animal burrows in arid desert soil (Kollath et al., 2019; Taylor and Barker, 2019). The genus has a broad distribution in both North and South America (Barker et al., 2012). In the United States alone, these fungi are estimated to cause disease in 350,000 individuals each year (Chiller, 2019). Disease burden in other regions is poorly documented; however, case rates are increasing across the new world (Alvarado et al., 2018; Freedman et al., 2018; Laniado-Laborin et al., 2019). Coccidioidomycosis (Valley fever) can result in a broad range of clinical symptoms. Respiratory infection is the most common, resulting in asymptomatic, acute, or chronic pneumonia. Dissemination occurs in rare cases (1–5%), infecting organs, bones, and the central nervous system (Galgiani et al., 2000, 2016; Ampel, 2015). As with other fungal infections, there is no vaccine and treatment options are limited to triazoles or polyenes (Thompson et al., 2019).

Disease prevention is difficult because natural or human caused disruption of the soil can cause the fungal propagules to become airborne, which can be inhaled by a susceptible mammal. In the environment, these arthroconidia germinate as polarized filamentous mycelia. Exposure to the host respiratory system triggers fungal morphogenesis to the host-specific lifecycle. Unknown factors cause isotropic swelling into large parasitic structures called spherules. These spherules swell over the course of about 5 days at which point they rupture releasing ∼100 endospores; and each of these endospores can develop into another rupturing spherule. Endosporulation is a fundamental step in host colonization as it allows for exponential increase in fungal burden approximately every 5 days (Drutz and Huppert, 1983; Cole and Hung, 2001).

Endosporulation is a key step in coccidioidomycosis; however, this stage is not well understood. *Coccidioides* is the only dimorphic fungal pathogen known to undergo this cyclical release of spores within a host (Sil and Andrianopoulos, 2014; Van Dyke et al., 2019). Understandably, research has focused on the genes utilized to develop from arthroconidia into the spherule structure. Thus far, only one published study compared the global transcriptome between the environmental and parasitic lifecycle between the two species (Whiston et al., 2012). They found 1,880 genes up-regulated in the spherules structure, which were shared by both species. While insightful, of the top 15 differentially expressed genes, 10 were hypothetical proteins without predicted function. This challenge is common in *Coccidioides* research as ∼49% of the genome is not annotated, and function is often inferred by orthologs to other well studied fungi. However, this strategy is only helpful with shared characteristics. Because endosporulation is unique to *Coccidioides*, we sought to develop an alternative approach to identify genes required to complete this unique lifecycle.

**FIGURE 1:**
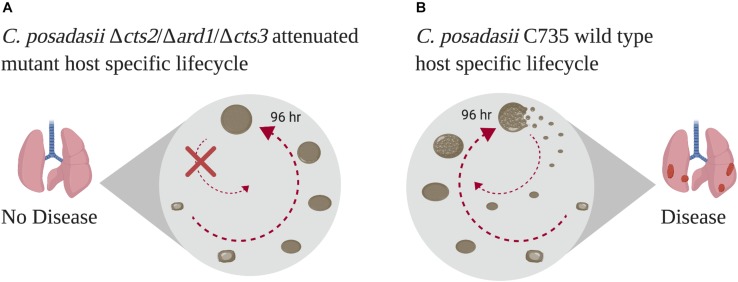
Comparison of mutant and parent lifecycles. The attenuated mutant **(A)** develops from arthroconidia into non-rupturing spherules that do not contain endospores. **(B)** In contrast, the wild-type strain begins to develop endospores around 96 h. These structures rupture near 120 h, releasing endospores which individually develop into mature, rupturing spherules every 120 h. Illustration created with BioRender.

To accomplish this, we utilized a mutant strain of *C. posadasii* Δ*cts2*/Δ*ard1*/Δ*cts3* which develops into sterile spherules that are unable to endosporulate (Figure 1; Xue et al., 2009). Chitinase 2 (*cts2*) and chitinase 3 (*cts3*) were selected by the authors based on real time PCR analysis of all chitinase gene activity during spherule remodeling and endospore formation. During cts3 disruption the open reading frame of a contiguous gene, D-arabinotol-2-dehydrogenase (*ARD1*) was impacted. In the study, the authors show that individual mutants Δ*cts2* or Δ*ard1*/Δ*cts3* retained the ability to endosporulate and cause disease. However, the combination of Δ*cts2*/Δ*ard1*/Δ*cts3* deletion prevented endosporulation and subsequently attenuation. The molecular determinants involved in the lack of endospore formation or release remains elusive. We compared growth of this mutant to the wild-type parent strain as a method of filtering through the previously identified differentially regulated transcripts based on the non-endosporulating phenotype. Consequently, this study focused on the 96 h time point which is when endospores are forming, and the cell wall is remodeling in preparation for spherule rupture (Cole and Hung, 2001). Additionally, this was the time point investigated in the study mentioned above comparing the transcriptome of environmental and parasitic stages of the wild-type parent, *C. posadasii* C735 (Whiston et al., 2012). Therefore, we compared the global transcriptome of the mutant strain, Δ*cts2*/Δ*ard1*/Δ*cts3*, to its wild-type parent strain C735 to define gene regulation during this crucial stage in disease establishment during a *Coccidioides* infection.

## Results

The pathways that regulate spherule development and survival of *Coccidioides* within a host are complex. The attenuated mutant strain Δ*cts2*/Δ*ard1*/Δ*cts3* provides an appropriate strategy for the reverse genetic studies used here. This strain is capable of creating the parasitic structure yet lacks necessary signaling pathways that allow for endospore formation and release (Figure 1). Therefore, the transcriptional profile of wild-type *C. posadasii* strain C735 and the attenuated mutant strain was compared to identify genes that are active in a key stage of disease establishment. Read data was generated by collecting 96 h spherules and mycelia from the attenuated strain of *Coccidioides*, Δ*cts2*/Δ*ard1*/Δ*cts3*, using growth conditions from the previously published experiment with the wild-type strain (Whiston et al., 2012). The raw reads for the wild-type parent strain were retrieved and subject to *de novo* analysis using the TopHat2/Cufflinks package alongside the newly generated mutant reads. This package uses two normalization parameters. Read counts are normalized based on the total reads obtained per sequencing run, which account for differences in sequencing runs. Additionally, within sample read counts are normalized based on total transcript length to avoid long transcripts accumulating more counts than short ones. The individual replicates for each condition had a high degree of similarity for each sample in terms of total transcript density and expression (Supplementary Figure 1). The transcripts clustered by lifecycle rather than strain (Supplementary Figure 2). Together these results indicate a high degree of similarity between individual replicates and data sets. For the full data set see Supplementary Table 1.

### Comparison of Wild-Type and Mutant Lifecycles; Mycelia Versus Spherules

**FIGURE 2:**
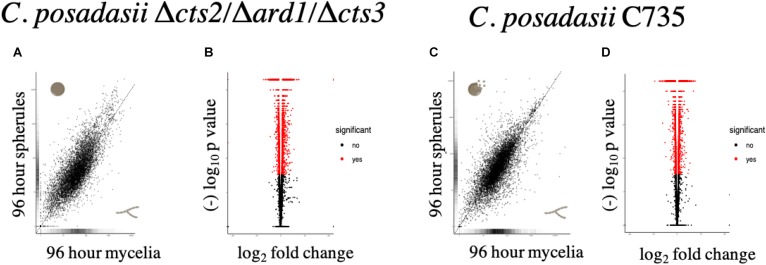
Comparison of global transcriptome and differentially expressed genes measured between 96-h spherules and mycelia for each strain. **(A,C)** The global expression pattern between spherules and mycelia are similar between strains. Transcripts that fall on the line are equally expressed in both cell types. Distance from the line indicates increased transcript abundance for spherules (y-axis) or mycelia (x-axis). **(B,D)** Using an FDR *p* < 0.05 there were 4,646 differentially expressed genes (2,409 up-regulated) in the mutant strain and 4,020 differentially expressed genes (2,118 up-regulated) in the parent strain.

**FIGURE 3:**
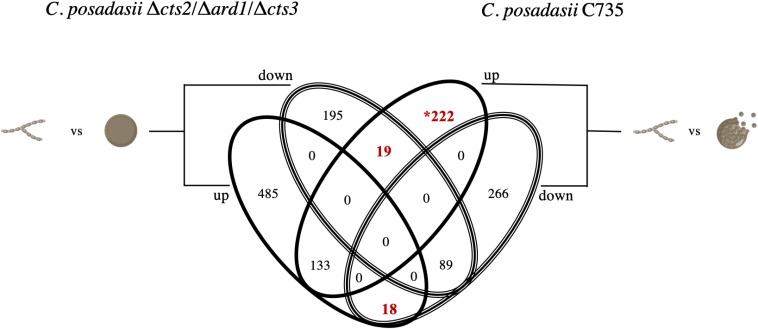
Venn diagram of differentially expressed genes between mycelia and spherules for each strain, respectively. Diagram shows genes that change expression in spherules compared to mycelia for the wild-type and mutant strain. Genes were included if expression was significantly (FDR *p* < 0.05) up-regulated (solid oval) or down-regulated (striped oval) twofold or more. Of interest are 259 gene transcripts that in the mutant deviate from the pattern of the wild-type parent: 19 genes up-regulated in the wild-type parasitic lifecycle and down in the mutant, 18 down-regulated in the wild-type parasitic lifecycle and up in the mutant, and 222 up regulated only in the wild-type lifecycle. Of these 259 genes, the asterisk (*) indicates that seven are unique to *Coccidioides*.

Using transcript data, we searched for deviations from the wild-type transcriptional profile using two approaches. First, the differential expression between lifecycle stages: the environmental (mycelia) versus parasitic (spherule) was quantified, for each strain respectively. The Δ*cts2*/Δ*ard1*/Δ*cts3* is derived from *C. posadasii* C735; therefore, the transcriptional pattern between lifecycles for each isolate was similar (Figures 2A,C). In the mutant strain, there were a total of 4,646 significantly differentially expressed genes, 2,423 were up-regulated and 2,223 down-regulated in 96 h spherules [False Discovery Rate (FDR), *p* < 0.05] (Figure 2B). In comparison, the parent strain had a total of 4,020 significantly expressed genes, 2,118 of which are up-regulated and 1,902 down-regulated in 96 h spherules (FDR, *p* < 0.05) (Figure 2D). Investigations were restricted to transcripts that are up or down-regulated twofold or greater between lifecycle stages for each strain respectively (FDR, *p* < 0.05). A Venn diagram was used to visualize similarity and differences between wild-type and mutant gene expression patterns (Figure 3; Oliveros, 2007). Genes from the Venn diagram are listed in Supplementary Table 2.

### Acquisition of Iron and Other Metal Co-factors Is Key to Preparation of Wild-Type Spherule Remodeling or Endosporulation

**FIGURE 4:**
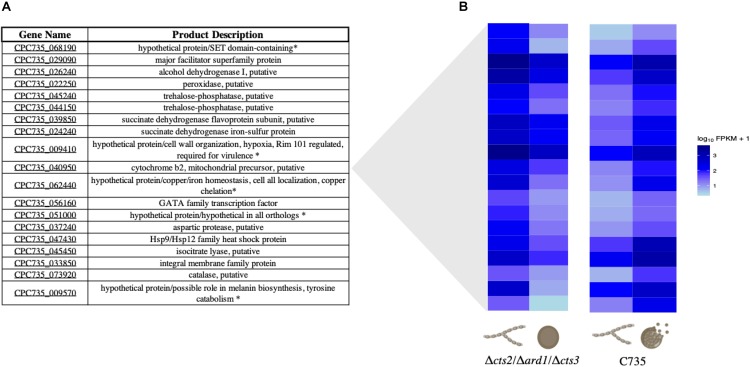
Gene description and heatmap of 19 improperly down-regulated genes. When comparing wild-type mycelia to spherules, these genes are significantly up-regulated. However, they are down-regulated in the mutant spherules compared to mycelia. **(A)** Table of the 19 genes and product descriptions, some of which have been shown to be important in stress response, pH, and metal ion acquisition in other fungi. When genes were annotated as hypothetical the asterisk (*) indicates ortholog function in other fungi. **(B)** Heatmap visualization in log_10_FPKM of the opposing expression patterns for wild-type and mutant lifecycle. A darker color indicates increased expression while lighter indicates decreased expression measured in log_10_ FPKM.

**FIGURE 5:**
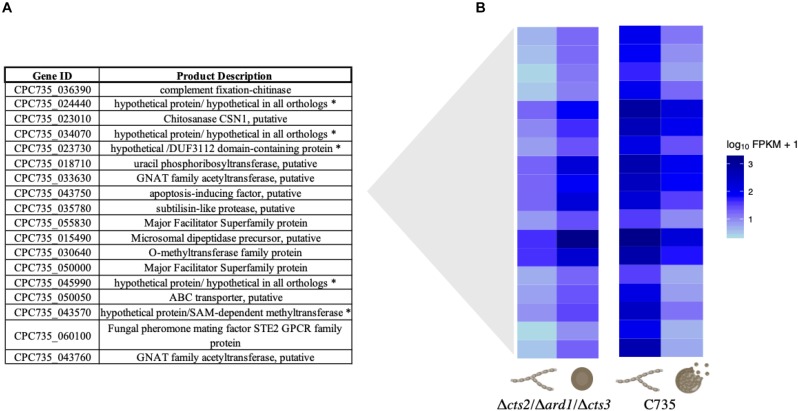
Gene description and heatmap of 18 improperly up-regulated genes. When comparing wild-type mycelia to spherules, these genes are significantly down-regulated. **(A)** Table showing gene descriptions, some of which are involved with chitin remodeling, mating pheromones/quorum sensing, and programmed cell death. When genes were annotated as hypothetical the asterisk (*) indicates ortholog function in other fungi. **(B)** Heatmap visualization in log_10_FPKM of the opposing expression patterns for wild-type and mutant lifecycles. A darker color indicates increased expression while lighter indicates decreased expression measured in log_10_ FPKM.

When searching for shared or disparate expression patterns between strains, 19 transcripts were identified that are up-regulated in the *C. posadasii* C735 parasitic lifecycle and down-regulated in *C. posadasii* Δ*cts2*/Δ*ard1*/Δ*cts3* (Figure 4). The transcriptional pattern of C735 can give insight into wild-type gene regulation during infection. In contrast, mutant deviations from the wild-type transcriptional pattern may represent genes necessary during endospore formation that are receiving improper signals or responding inappropriately during spherule remodeling. Of these genes, five are annotated as hypothetical proteins; however, function was inferred for these genes using annotated orthologs (Figure 4). Overrepresented biological categories were iron acquisition, metal ion homeostasis, and response to oxidative stress (Supplementary Table 3). In the wild-type expression profile, a GATA transcription factor was significantly up-regulated in spherules but down-regulated in the mutant. This gene is conserved across fungi and implicated in iron homeostasis, siderophore biosynthesis, and morphogenesis (Oberegger et al., 2001; Hwang et al., 2008; Gauthier et al., 2010; Attarian et al., 2018). These data suggest that the acquisition of iron and other metals in *Coccidioides* is a key cellular process in preparation for spherule remodeling or endosporulation. Interestingly, these processes are impacted by the dysregulation of chitinases, as observed in the mutant strain expression profile. Similarly, there were 18 transcripts that are down-regulated in the wild-type parasitic lifecycle (mycelia versus spherule) while up-regulated in the mutant (Figure 5). These transcripts are specific to chitin remodeling, secreted proteins, and pheromone sensing. GO terms are enriched for catabolism, membrane transport, and programmed cell death (Supplementary Table 4).

### Nitrogen Recycling, Mitochondrial Respiration and Heat Shock Proteins Are Key Transcripts Up-Regulated in Wild-Type Spherules Compared to Mycelia

Next, the 222 genes that are up-regulated in the wild-type parasitic lifecycle, but not in the mutant lifecycle (Figure 3) were investigated. We predict that genes up-regulated in both strains are required for spherule growth and maintenance. In contrast, genes that are only up-regulated in the wild type at 96 h may be involved in the cellular signals that lead to endospore formation, or spherule rupture. GO terms suggest that the wild-type strain exhibits transcripts specific to nitrogen assimilation or metabolism (Table 1). Out of the 222 genes mentioned, 102 are annotated as hypothetical proteins, with orthologs in many other fungi (Supplementary Table 5). This indicates that *Coccidioides* uses conserved genes to complete the parasitic lifecycle, at least in part. In contrast, eight genes are currently identified as exclusive to the *Coccidioides* genus. Although other pathogenic fungi undergo host-specific morphogenesis, endospore formation and release is a unique characteristic of *Coccidioides*. With this in mind, the amino acid sequences for each gene were used to search for conserved protein domains using several prediction algorithms; NCBI conserved domains search, EggNOG Mapper and InterProScan (Supplementary Table 6). Several of the hypothetical proteins are suggested to have protein kinase-like or phosphotransferase-like domains. Thus, these unknown genes could potentially participate in signal cascades modifying other proteins by the addition of a phosphate group. We determined that one gene, CPC735_012730, is similar to pyoverdine/dityrosine biosynthesis protein which is a precursor to the ascospore cell wall of *Saccharomyces cerevisiae* (Briza et al., 1994). This conserved architecture was identified by all three of the prediction algorithms utilized. For two of these *Coccidioides*-specific genes, the prediction methods used in this study were unable to derive any putative function. Nevertheless, the transcriptional activity of these genes in the wild-type lifecycle makes them interesting candidates for future investigations. We further interrogated this list of 222 genes for transcripts exhibiting the highest fold change in wild-type mycelia to spherules. Of interest is Hsp20, a heat shock protein which is likely involved in chaperoning proteins in response to heat stress. Curiously, this gene is expressed sixfold higher in the wild-type parasitic lifecycle while there is little expression change in the mutant from mycelia to spherules (Table 2). We predict that this chaperone protein is related to spherule structural remodeling, which is impaired in the mutant. Not surprisingly, other genes with known function in this highly up-regulated group are implicated in stress response, or in mitochondrial function. These genes do not display the same transcriptional activity in the mutant suggesting that they are important in wild-type spherules during the time point when spherules remodel, form endospores and subsequently prepare for rupture and dissemination.

**TABLE 1:** Significantly enriched biological processes up-regulated in wild-type spherule compared to mycelia.

GO ID	GO term	Percent of bkgd genes in your result	Fold enrichment	Odds ratio	Benjamini	Bonferroni	Organism
GO:0120029	Proton export across plasma membrane	100	36.51	Infinity	5.64E-03	2.26E-02	A. f f293
GO:0120029	Proton export across plasma membrane	100	27.97	Infinity	4.80E-05	3.36E-04	A. n 1015
GO:0140115	Export across plasma membrane	100	27.97	Infinity	4.80E-05	3.36E-04	A. n 1015
GO:0008272	Sulfate transport	75	20.98	81.99	2.65E-03	3.71E-02	A. n 1015
GO:0072348	Sulfur compound transport	75	20.98	81.99	2.65E-03	3.71E-02	A. n 1015
GO:0042126	Nitrate metabolic process	57.1	15.98	36.59	1.08E-03	1.08E-02	A. n 1015
GO:0042128	Nitrate assimilation	57.1	15.98	36.59	1.08E-03	1.08E-02	A. n 1015
GO:0071941	Nitrogen cycle metabolic process	50	13.99	27.44	1.75E-03	2.10E-02	A. n 1015
GO:2001057	Reactive nitrogen species metabolic process	50	13.99	27.44	1.75E-03	2.10E-02	A. n 1015
GO:0006979	Response to oxidative stress	47.4	13.25	25.23	2.91E-07	1.16E-06	A. n 1015
GO:0006995	Cellular response to nitrogen starvation	31.3	11.41	16.43	9.37E-03	5.62E-02	A. f f293
GO:0043562	Cellular response to nitrogen levels	31.3	11.41	16.43	9.37E-03	5.62E-02	A. f f293
GO:0016999	Antibiotic metabolic process	13	4.75	5.52	1.15E-03	3.44E-03	A. f f293
GO:0055085	Transmembrane transport	8.3	2.32	2.98	8.72E-09	8.72E-09	A. n 1015
GO:0055114	Oxidation-reduction process	6.5	2.38	2.76	5.96E-05	5.96E-05	A. f f293
GO:0055085	Transmembrane transport	6.5	2.37	2.73	9.96E-05	1.99E-04	A. f f293
GO:0006810	Transport	7.2	2.02	2.54	2.91E-07	1.04E-06	A. n 1015
GO:0051234	Establishment of localization	7.2	2.02	2.54	2.91E-07	1.09E-06	A. n 1015
GO:0051179	Localization	7.1	1.99	2.51	3.43E-07	1.71E-06	A. n 1015
GO:0055114	Oxidation-reduction process	6.1	1.7	1.99	6.50E-04	5.20E-03	A. n 1015

*Terms are based on *A. niger* (A.n 1015) and *A. fumigatus* (A.f f293). Over represented biological processes suggest nitrogen utilization, toxin production, and mitochondrial related genes demonstrate increased activity during cellular preparation for endospore release.*

**TABLE 2:** Transcripts that are up-regulated fivefold or more in wild-type spherules compared to mycelia and not in the mutant.

Gene ID	Product description	Ortholog group	*C. posadasii* C735 log2 fold change*	*C. posadasii* Δ*cts2*/Δ*ard1*/Δ*cts3* log2 fold change*
CPC735_010730	Hypothetical protein/Fungal dehydrin-like protein that plays a role in oxidative, osmotic and pH stress responses**	OG5_155947	7.68551	0.885797
CPC735_018870	Hypothetical protein/conserved hypothetical**	OG5_144599	6.50284	−0.574354
CPC735_037080	Hypothetical protein/conserved hypothetical**	OG5_180711	5.71183	1.98425
CPC735_047390	Hsp20/alpha crystallin family protein	OG5_126935	6.96036	0.716718
CPC735_053080	Oxidoreductase, short chain dehydrogenase/reductase family protein	OG5_128170	5.03474	0.539224
CPC735_070700	Hypothetical protein/mitochondrial integral membrane protein**	OG5_139306	5.32416	0.860862
CPC735_070990	Hemerythrin family protein	OG5_139259	5.84751	−1.3612

Gene ID, product description, ortholog group and fold change are listed for both wild-type and mutant comparisons. These transcripts are associated with heat or stress tolerance and mitochondrial respiration. Asterisk (*) indicates fold change between mycelia and spherules, (**) indicates ortholog function.

### Comparison of Transcript Abundance Between Wild-Type and Mutant Spherules Identifies Several *Coccidioides* Specific Hypothetical Proteins

**FIGURE 6:**
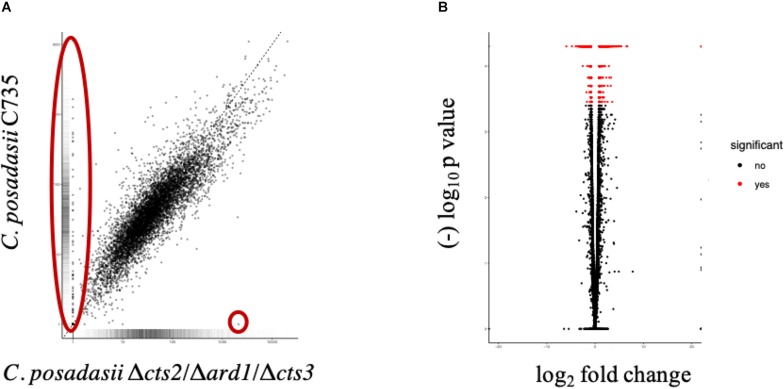
Comparisons of global transcriptome and differentially expressed genes measured between 96 h spherules for parent (C735) and mutant (Δ*cts2*/Δ*ard1*/Δ*cts3*). We observed a subset of genes that are expressed (FDR, *p* < 0.001) in wild-type spherules and inactive in the mutant (**A**, large oval) compared to the attenuated mutant. These genes may be related to endospore formation or release. Hygromycin resistance cassette is expressed in the mutant only (**A**, small circle). **(B)** There are 2,018 genes differentially expressed (FDR, *p* < 0.001) between spherules, 151 are upregulated twofold or more.

**FIGURE 7:**
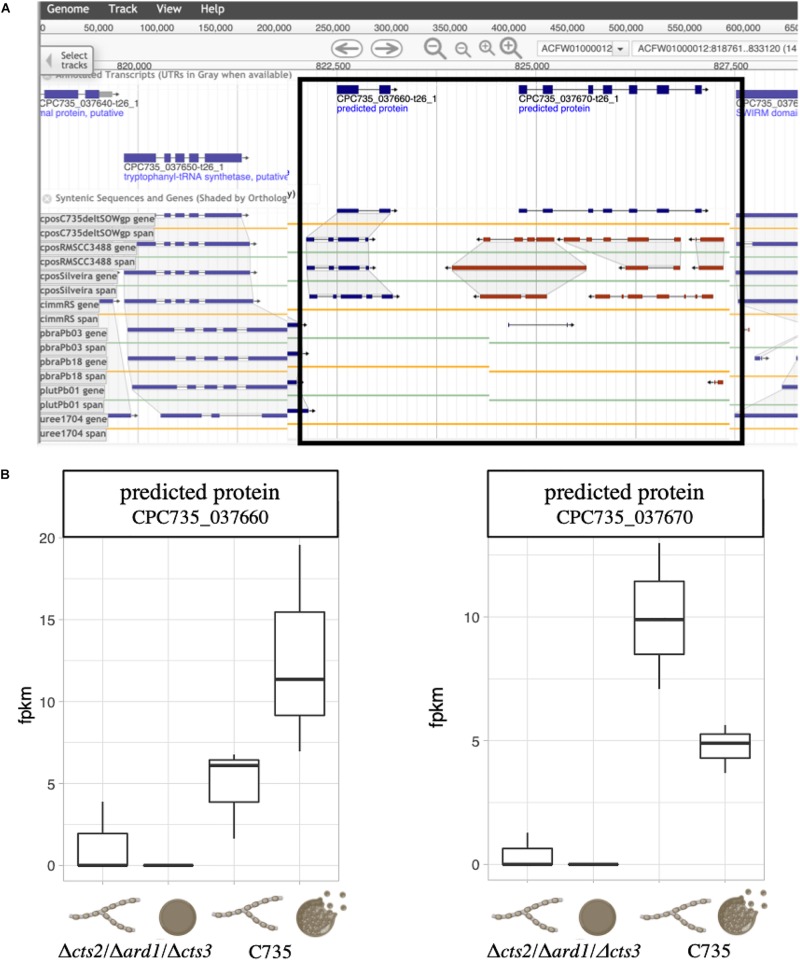
Location and expression of putative morphogenesis related genes. **(A)** The predicted proteins are located adjacent to one another and are unique to *Coccidioides*. **(B)** In the *C. posadasii* C735 strain, the gene CPC735_037660 is up-regulated in wild-type spherules and CPC735_037670 is down-regulated. In the non-endosporulating mutant spherules, no transcripts were detected and low levels were detected in the mycelia phase.

**FIGURE 8:**
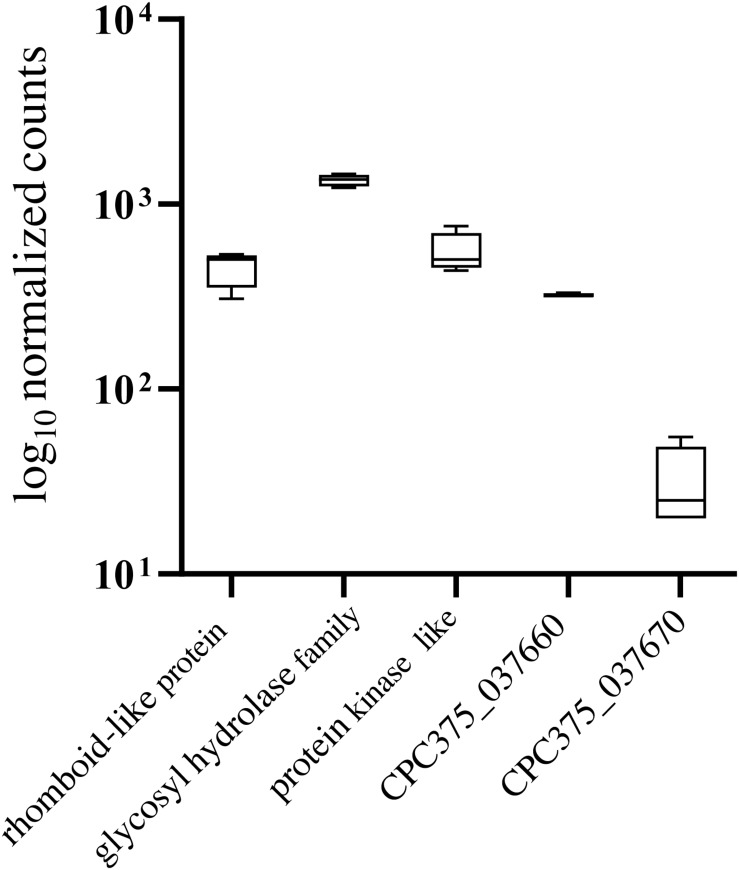
*In vivo* expression in a murine model. Normalized counts of housekeeping genes and candidate genes at 96 h using NanoString technology. The genes CPC735_037660 and CPC735_037670 are active at 96 h in the host tissue in both *C. posadasii* strain C735 and *C. posadasii* Silveira. CPC735_037660 shows higher activity than CPC735_037670 similar to the *in vitro* observations.

As a second approach, we compared transcript expression specifically between wild-type and mutant spherules (Figure 6A). Because different sequencing platforms were used to obtain reads (GAII, MiSeq), significance comparisons were further restricted to FDR corrected *p* < 0.001 to reduce the possibility of false positives. There were 2,018 significantly differentially expressed genes 151 of which were up-regulated twofold or more in the wild-type spherules as compared to the mutant spherules (Figure 6B). GO terms indicate enrichment of biological processes specific to transport across the membrane and response to oxidative stress (Supplementary Table 7). Of specific interest, cell division control (CPC735_003020) and circadian rhythm (CPC735_054180) proteins were significantly up-regulated in wild-type spherules compared to mutant spherules. These genes could be part of the signaling pathways responsible for endospore formation in wild-type *Coccidioides*. From the list of 151 genes, 77 are annotated as hypothetical proteins with orthologs to other fungi. However, six are specific to *Coccidioides*. For each gene, three prediction methods (NCBI conserved domains search, EggNOG Mapper and InterProScan) were used to query the protein sequence to search for a conserved architecture. These strategies were unable to detect conserved domains, or similar sequences for any of these genes (Table 3). Without conserved domain structure it is difficult to speculate function of these unknown gene transcripts. However, the observed expression patterns would suggest these genes are important during spherule remodeling and endospore formation. Within this list of unknown proteins, there were a subset that are active in wild-type spherules and not active in the mutant spherules (Table 3). Two of these genes, CPC735_037660 and CPC735_037670, are adjacent to each other, and are unique to *Coccidioides* (Figure 7A). The wild-type expression pattern of the gene set reveals that 037660 demonstrates increased transcript abundance in spherules and its genomic neighbor 037670 is active during hyphal growth. Intriguingly, both transcripts are absent in mutant spherules, further suggesting a specific association with morphogenesis in spherules (Figure 7B). To ensure that these genes are operational during infection, a custom set of RNA probes was employed. These transcripts were detected *in vivo* in a murine model at the same time point, suggesting that these genes are active in a host environment (Figure 8).

**TABLE 3:** Six *Coccidioides* specific hypothetical proteins that are significantly up regulated in the wild-type spherules compared to mutant spherules.

Gene description			Conserved domains			Transcript abundance		
Gene ID	Product description	Ortholog group	NCBI	InterproScan	EggNOG mapper	Δ*cts2*/Δ*ard1*/Δ*cts3* Spherules FPKM	C735 wild-type spherules FPKM	FDR *p*-value
CPC735_025360	Hypothetical protein	OG5_cpos CPAG_03276	No hits	No hits	No hits	16.4916	94.7691	0.000150527
CPC735_030120	Hypothetical protein	OG5_cpos CPC735_030120	No hits	No hits	No hits	0	256.854	0.000150527
CPC735_037660	Predicted protein	OG5_223849	No hits	No hits	No hits	0	12.6279	0.000150527
CPC735_037670	Predicted protein	OG5_cpos CPC735_037670	No hits	No hits	No hits	0	4.74221	0.000150527
CPC735_040990	Hypothetical protein	OG5_cpos CPAG_00370	No hits	No hits	No hits	8.62265	87.1783	0.000150527
CPC735_073570	Hypothetical protein	OG5_223158	No hits	No hits	No hits	14.7823	59.9105	0.000150527

*Results of *in silico* predictions based on amino acid sequence are listed. The lack of expression in the mutant strain might indicate improperly signaling due to gene deletion. These *Coccidioides* specific genes are likely important to spherule remodeling.*

### Differential Volatile Metabolome of Δ*cts2*/Δ*ard1*/Δ*cts3* Compared to Parent Strain Reveals Unique Compounds Produced by Wild-Type *C. posadasii*

Several of the genes that are differentially regulated in *C. posadasii* C735 versus Δ*cts2*/Δ*ard1*/Δ*cts3* are predicted to be involved in key metabolic processes, such as nitrate and sulfate transport and metabolism and detoxification of alcohols and reactive oxygen species, and therefore we compared the metabolomes of the wild-type and mutant strain. Our group is focused on developing breath-based biomarkers of *Coccidioides* infections, and therefore analyses were restricted to metabolites that are detected in the gas phase. Solid phase microextraction (SPME) and comprehensive two-dimensional gas chromatography – time-of-flight mass spectrometry (GC × GC-TOFMS) were used to collect and analyze the volatile organic compounds (VOCs) produced by 96 h spherules of C735 and attenuated strain. After data alignment and removal of chromatographic artifacts, 522 VOCs were detected across the nine samples, which included three biological replicates of each strain and three media blanks.

**FIGURE 9:**
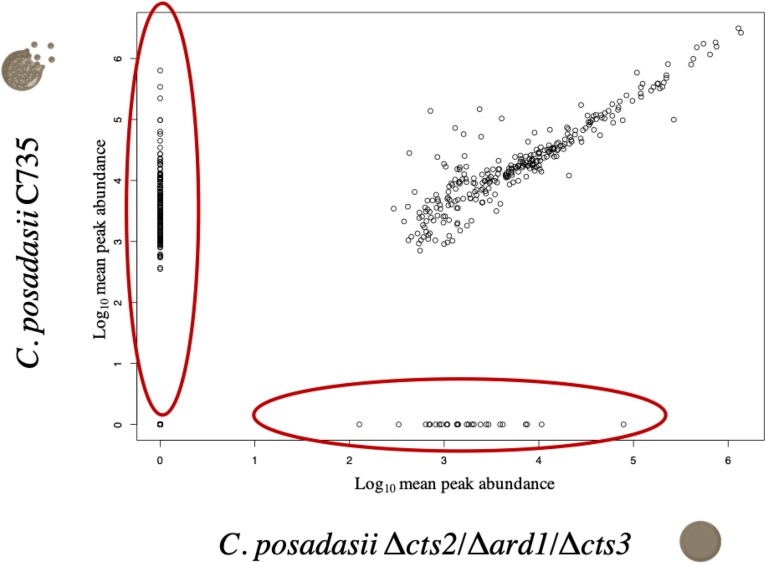
Comparisons of the volatile metabolomes of 96-h spherules for *C. posadasii* strain C735 and attenuated mutant (Δ*cts2*/Δ*ard1*/Δ*cts3*). Individual circles represent log-transformed mean peak abundance across three biological replicates. Compounds in large red oval on the y-axis are the 130 significant VOCs uniquely produced by C735 (*n* = 40; FDR *p* < 0.05) or uniquely consumed by Δ*cts2*/Δ*ard1*/Δ*cts3* (*n* = 90; FDR *p* < 0.05). Compounds in large red oval on the x-axis are the 28 VOCs uniquely consumed by C735 (*n* = 12; *p* < 0.05) or uniquely produced by Δ*cts2*/Δ*ard1*/Δ*cts3* (*n* = 16; *p* < 0.05).

A comparison of the *C. posadasii* C735 and Δ*cts2*/Δ*ard1*/Δ*cts3* VOCs shows these two strains are quite different, with an almost fivefold increase in the number of metabolites detected in wild-type *C. posadasii* C735 cultures (Figure 9). Of the total metabolome, about a third (*n* = 158) of the VOCs were highly reproducible across biological replicates and either uniquely produced (present in one strain but absent in the other strain and media blanks) or uniquely consumed (absent in one strain but present in the other strain and media blanks) by one strain or the other, these were the focus for further characterization (Supplementary Table 8). The unique production of VOCs was dominated by *C. posadasii* C735, with 40 VOCs compared to 16 by Δ*cts2*/Δ*ard1*/Δ*cts3*, and the converse was true for metabolite consumption, with 90 compounds uniquely consumed by Δ*cts2*/Δ*ard1*/Δ*cts3* versus 12 by C735. A Wilcoxon Rank Sum test with Benjamini and Hochberg FDR correction was performed on these 158 VOCs, and all 40 volatiles uniquely produced by *C. posadasii* C735 and all 90 volatiles uniquely consumed by *C. posadasii*, Δ*cts2*/Δ*ard1*/Δ*cts3* were found to be significant (*p* < 0.05) (Figure 9). Based upon mass spectral and chromatographic data, putative identities were assigned to 13 VOCs, the majority of which were aromatic, heteroaromatic, or aldehyde compounds (Supplementary Table 8). For the unnamed VOCs, chemical classifications were assigned to 58 of them based on a combination of mass spectral and chromatographic characteristics. The remaining 87 VOCs are classified as unknowns, due to a lack of mass spectral or chromatographic data of sufficient quality for identification.

The metabolomics data broadly reflect the same trends as the transcriptomics data, with higher proportions of metabolites being found specifically in the C735 cultures (Figure 9 and Supplementary Table 8). The metabolomics data also provide a second window into the physiology of these two strains by quantifying the balance of metabolic production versus consumption by comparing VOC abundances in the fungal cultures versus the media blanks. Of the 158 uniquely produced or consumed VOCs, C735 has a threefold net production, while Δ*cts2*/Δ*ard1*/Δ*cts3* has a fivefold net consumption. These data would suggest that the mutant is able to consume the nutrient resources it would require for spherule remodeling or endospore formation, but the downstream metabolic pathways for this physiological transformation are not activated.

The comparative transcriptomics of C735 and Δ*cts2*/Δ*ard1*/Δ*cts3* predicted lower peroxidase, catalase, and alcohol dehydrogenase activity in the mutant, which we hypothesized would yield higher relative abundances of peroxides and lower aldehydes in the mutant versus wild type. Further, with higher levels of sulfur and nitrate transport and metabolism genes in the wild type, we would predict differences in heteroaromatic, nitrogen-containing, and sulfur-containing VOCs between wild type and mutant. Some of the metabolomics data support these hypotheses, while other data refute them. For instance, fewer aldehydes were produced by Δ*cts2*/Δ*ard1*/Δ*cts3*, but also fewer peroxides. However, the very large imbalance in the numbers of VOCs produced by C735 and Δ*cts2*/Δ*ard1*/Δ*cts3*, or consumed by these two strains inhibit our ability to robustly test these pathway-specific hypotheses.

### Transmission Electron Microscopy Demonstrates Impaired Cell Wall and Internal Remodeling Activity in Δ*cts2*/Δ*ard1*/Δ*cts3* Spherules Compared to C735 Spherules

**FIGURE 10:**
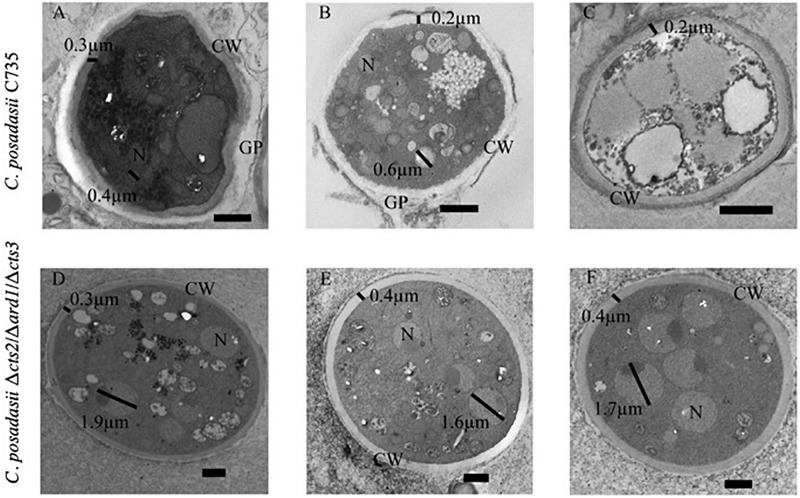
Transmission Electron Microscopy Images of *C. posadasii* strain C735 and △cts2/△ard1/△cts3 spherules. Wild-type spherule with thick, irregular cell wall (CW) and sloughing glycoprotein (GP) shell that are visible after 96-h development *in vitro* **(A)**. Small nuclei (N), irregular cell wall, sloughing glycoprotein were consistently visible in wild-type spherules **(B)**. Cross-walls indicating the possible commencement of endospore formation were visible in wild-type spherules after 96-h development **(C)** but not in the mutant. In general, the Δ*cts2*/Δ*ard1*/Δ*cts3* cell wall is rounder and more consistent **(D)** than the wild type. Glycoprotein coating is less visible in mutant spherules **(E)** than in wild type. Nuclei are larger, more distinct and visible in spherules of the mutant **(F)** than of the wild type. Black bars in the bottom right corner of images represent 1 μm.

Finally, we used transmission electron microscopy (TEM) to view the internal landscape of 96 h Δ*cts2*/Δ*ard1*/Δ*cts3* and C735 spherules to look for evidence of phenotypic differences at this time point (Figure 10). The exterior of the wild-type spherules possesses loose layers of material, likely glycoproteins which have previously been reported to consistently shed from the cell wall (Figures 10A,B)(Hung et al., 2002). In *C. posadasii* C735 spherules, there were thick cell walls, and irregular shaped spherules which would indicate active cell wall remodeling (Figures 10A–C). This phenotype is consistent with previous reports and precedes internal segmentation (Huppert et al., 1982). Additionally, there were a variety of internal structural patterns, indicating active spherule remodeling and evidence of initiation of cross wall formation (Figure 10C). In contrast, the Δ*cts2*/Δ*ard1*/Δ*cts3* spherule development appeared partially arrested, with little cell wall or internal structure variation through-out all images (Figures 10D–F). We did not observe evidence of glycoprotein sloughing, rather the cell walls were consistently even in thickness and overall shape. The cell wall of the mutant strain appears moderately thicker than the wild type. Interestingly, the mutant strain exhibited abnormally large nuclei compared to the wild type (Figures 10D–F and Supplementary Table 9). These images suggest that the mutant strain may undergo DNA replication, but not nuclear division. In wild-type strains, free nuclear division occurs prior to internal segmentation and endospore formation (Huppert et al., 1982). This phenotypic evidence implies that gene deletion impairs both cell wall and internal remodeling at this time point.

## Discussion

For this study, the transcriptional and volatile profile of a non-endosporulating attenuated strain, Δ*cts2*/Δ*ard1*/Δ*cts3*, of *Coccidioides posadasii* was compared to its pathogenic parent strain, C735. The mutant strain develops into non-rupturing spherules and is incapable of causing disease in a murine model (Xue et al., 2009). This phenotype was obtained by deletion of two chitinase genes (*cts2*, *cts3*), which were targeted due to their increased expression in spherules. Interestingly, the investigation revealed that independent mutants Δ*cts2*/Δ*ard1* and Δ*cts3* demonstrated delayed endosporulation and could still cause disease. Attenuation was achieved only during the removal of both chitinase genes (Xue et al., 2009). Chitinases are responsible for remodeling the cell wall; however, why the combination results in attenuated spherules is not understood. Consequently, the gene regulation related to endospore formation, spherule remodeling and subsequently disease establishment might be elucidated through comparing the wild-type and mutant.

We observed 252 gene transcripts that are evolutionarily conserved and improperly expressed by the mutant strain. Of these, 18 are improperly down-regulated and 19 improperly up-regulated when comparing the mutant lifecycle (96-h spherules to mycelia) to the wild-type lifecycle (96-h spherules to mycelia). Additionally, 215 conserved transcripts (out of 222) are only up-regulated twofold or more in wild-type spherules (Figure 3). We can take advantage of these universal fungal adaptations to predict how these genes function in *Coccidioides*. For instance, upon exposure to the host environment, dimorphic fungi must initiate niche specific morphogenesis (Sil and Andrianopoulos, 2014; Boyce and Andrianopoulos, 2015; Van Dyke et al., 2019). While all fungal pathogens face the challenge of acquiring nutrients and avoiding immune system detection, the cell wall serves as an interface between the fungal cell and host milieu. The interaction between the cell wall, cell membrane, and mitochondrial function is key to this intricate response, which we also observed at a transcriptional level in our study (Verma et al., 2018; Koch and Traven, 2019).

One gene showing improper response in the mutant strain is a predicted Hsp9/Hsp12 family heat shock protein. This highly conserved gene is expressed during stress response, changes in pH, and may increase cell membrane stability (Sheth et al., 2008; Welker et al., 2010; Fu et al., 2012). Importantly, heat shock proteins have been shown to be up-regulated in other fungal pathogens such as *Cryptococcus neoformans* at low temperatures and *Candida albicans* at high temperatures (Steen et al., 2003; Fu et al., 2012). The function of this gene is unknown in *Coccidioides;* however, the abnormal down-regulation in the mutant strain could indicate that Hsp9/Hsp12 are chaperones for endospore related proteins, or increase the cell membrane stability prior to rupture.

Upon exposure to the mammalian environment (often the respiratory system), fungal pathogens must utilize potentially different carbon or nitrogen sources while competing with host cells for limited co-factors such as iron and copper (Marzluf, 1997; Chung et al., 2012; Braunsdorf et al., 2016; Bairwa et al., 2017). In many pathogenic fungi, genes related to trehalose biosynthesis have been implicated as critical in metabolism and stress response (Thammahong et al., 2017). In *Aspergillus*, deletion of tspA/B delayed germination and decreased virulence (Al-Bader et al., 2010). Interestingly, the authors observed thinner cell walls in tspA/B mutants, in contrast to our TEM observations. The Δ*cts2*/Δ*ard1*/Δ*cts3* strain down regulated trehalose-phosphotase transcripts where as they are up-regulated in the wild-type expression profile (Figure 4). This suggests that the trehalose-related pathways may affect the cell wall in both *Aspergillus* and *Coccidioides* but through different mechanisms. Another key nutrient, nitrogen, may be readily available in the environment and can become scarce inside the host. *A. fumigatus* senses nitrogen availability via a mitogen-activated protein (MAP) kinase pathway (Xue et al., 2004). Additionally, transcription factors (TFs) such as the highly conserved class of GATA TF’s activate alternative nitrogen processing pathways in the absence of preferred sources. These regulators are often highly expressed in both *in vitro* and *in vivo* infection conditions and are linked to fungal virulence (Oberegger et al., 2001; Gauthier et al., 2010; Lee et al., 2011; Chung et al., 2012; Hwang et al., 2012). In *C. neoformans*, the Gat1 TF regulates capsule formation (Lee et al., 2011). This same class of transcription factors has been shown to be essential for virulence in *C. albicans* (Limjindaporn et al., 2003). The GATA class of TFs are also associated with stimulating iron acquisition and host phase transition in *Blastomyces dermatitidis* (Gauthier et al., 2010). In our study, this GATA ortholog is improperly down-regulated compared to the pattern observed in the wild type (Figure 4).

In this study, metabolic dissimilarities were also detected in the uniquely produced and consumed volatile compounds between the parent and mutant strain. The Δ*cts2*/Δ*ard1*/Δ*cts3* demonstrated a fivefold consumption versus production of compounds that was not observed in the parent strain, indicating improper scavenging of resources; whereas, in the wild-type strain we observe the unique consumption of compound classes such as nitrogen containing, hydrocarbons, ethers, and alcohols. In addition, our RNAseq data demonstrate that transcripts for nutrient assimilation strategies specific to nitrogen and iron are up-regulated in 96-h wild-type spherules, but not in the mutant strain. Together these data implicate nitrogen and metal co-factors as necessary components during spherule structural remodeling to create or release endospores. Nitrogen containing functional groups were also among the uniquely produced VOCs in wild-type spherules. Previous studies have shown that rupturing spherules release ammonia (Wise et al., 2013) and urease (Mirbod-Donovan et al., 2006), which contribute to host damage and virulence. Further, it has been demonstrated that extracellular concentrations of ammonia begin to accumulate at 96 h peaking at 120 h, the time point just before spherule rupture (Wise et al., 2013). The lack of transcript abundance in the non-rupturing mutant highlights the importance of nitrogen recycling at this time point and subsequently disease establishment upon rupture.

Another crucial step in disease establishment is the ability of fungal organisms to avoid immune system detection and clearing (Erwig and Gow, 2016). Host cells respond to pathogen associated recognition patterns (PAMPs) located on the fungal cell wall (Latge, 2010). Consequently, the fungal cell wall is remodeled, a process controlled by the protein kinase c pathway (Munro et al., 2007; Gow et al., 2017; Koch and Traven, 2019). The masking of exposed ß-glucan, a key PAMP, is associated with cAMP-protein kinase A pathway (cAMP/PKA). This pathway is triggered by hypoxic conditions due to influx of immune cells or inflamed tissue (Pradhan et al., 2018; Duvenage et al., 2019). In *C. albicans*, chemical inhibition of respiration decreased ß-glucan masking and cell wall integrity (Pradhan et al., 2018; Duvenage et al., 2019) thus increasing immune system recognition and phagocytosis. In our data, mitochondria-associated genes, oxidation-reduction, cytochrome P450, and FAD binding proteins were all significantly up-regulated in the wild-type spherules compared to mycelia, but not in the same comparison for the mutant (Table 1 and Supplementary Table 6). We also observed more peroxide compounds in the VOCs produced by wild-type spherules versus the mutant. We suggest this is related to the deletion of *cts2*/*cts3* which impairs chitin remodeling and alters cell wall composition. This subsequently alters normal cell membrane communication and associated mitochondrial response.

We have previously shown that the exterior surface of these attenuated spherules is strikingly different to wild type at high resolution (Mead et al., 2019). In this study, we observed reduction in glycoprotein sloughing and a slight decrease in cell wall thickness in the mutant. The Δ*cts2*/Δ*ard1*/Δ*cts3* mutant was originally developed as a vaccine candidate and provides full protection to wild-type challenge in a murine model (Xue et al., 2009). Host protection is accomplished by a mixed Th1, Th2, and Th17 immune response (Xue et al., 2009; Hung et al., 2011). This is interesting given that previous studies have shown that the cell wall associated ß-glucan receptor dectin-1 is required for resistance to coccidioidomycosis. This receptor stimulates proinflammatory infiltrates through a Th1 and Th17 immune response in mice (Viriyakosol et al., 2005, 2013). To our knowledge, the ß-glucan composition of the attenuated strain has not been characterized. Our results suggest that deletion of the Δ*cts2*/Δ*ard1*/Δ*cts3* changes the cell wall structure which might alter ß-glucan masking. Our TEM images from this study further support this idea.

Lastly, in this study, we identified 14 *Coccidioides*-specific genes that are either inactive or improperly expressed by the mutant strain at this crucial time point. Seven were up-regulated twofold or higher in the wild-type 96-h spherules compared to mycelia, and but not in mutant spherules compared to mutant mycelia (Figure 3, Table 3 and Supplementary Table 6). We identified conserved architecture for some of these proteins. Based on this, these unknown proteins might be involved in kinase cascade activation or endonuclease activity. Furthermore, two genes were found to be inactive in the mutant spherules but active in wild-type spherules and expressed in host tissue. The close genomic proximity of these two genes suggests that they may be co-regulated in a *Coccidioides*-specific manner (Figure 7). We hypothesize that these two genes are crucial for spherule morphogenesis and endospore formation, which is necessary for proliferation within the host. In future studies, we hope to elucidate the role of these two *Coccidioides*-specific genes.

In summary, we compare the transcriptional and metabolic profiles of *C. posadasii* C735 and the attenuated mutant, Δ*cts2*/Δ*ard1*/Δ*cts3*. We reveal significant changes in both *Coccidioides*-specific transcripts involved in endospore formation, and those that are conserved among many fungal pathogens. We identified approximately 280 transcripts that are inappropriately regulated in the mutant strain with 14 specific to the *Coccidioides* genus. With the increase of fungal infections and emerging novel species, we suggest that comparing gene expression between a pathogenic parent and an attenuated mutant could be used to link phenotypic traits of interest to transcript specific pathways in many fungal systems. While each species may employ unique strategies, the challenges of survival within the mammalian host are shared. Attenuation of virulence can be achieved via several mechanisms; however, a shared pattern of transcriptional regulation may elucidate crucial conserved pathways for virulence, and suggest novel targets for drug development and vaccines.

## Materials and Methods

### Fungal Isolates and Growth Conditions

*Isolates:* The wild-type strain utilized was *C. posadasii* isolate C735 (ATCC 96335). The attenuated strain was derived from the clinical isolate C735 and was obtained through Biodefense and Emerging Infections (BEI Resources) repository, (NIAID, NIH: *Coccidioides posadasii*, Δ*cts2*/Δ*ard1*/Δ*cts3*, NR-166). *C. posadasii* isolates C735 and Silveira were also used in the pulmonary infections. All wild-type *Coccidioides* spp. strains are grown under biosafety level 3 containment.

*Saprobic growth conditions:* Arthroconidia were pipetted into a 250 ml vented baffle flask containing 50 ml of 2 × glucose yeast extract (2 × GYE; 2% glucose (VWR), 1% yeast extract (Difco) on a shaking incubator (Gene-Mate Orbital Shaker Mini) at 30°C for 96 h. Samples were grown in triplicate. Sterile media controls were treated in the same manner.

*Parasitic growth conditions:* Arthroconidia were pipetted into vented baffled flasks (VWR) on a shaking incubator (GeneMate Orbital Shaker Mini) at 90 rpm, incubated at 10% CO_2_, 39°C, at ambient oxygen (ThermoForma Series II water jacketed CO_2_/O_2_ incubator) in chemically defined modified Converse media (ammonium acetate 0.016 M (Sigma-Aldrich), KH_2_PO_4_ 0.0037 M (Amresco), K_2_HPO_4_ 0.003 M (Amresco), MgSO_4_ 0.0016 M (Amresco), ZnSO_4_ 1.25 × 10^–5^ M (Fisher Scientific), NaCl 2.4 × 10^–4^ M (Fisher Scientific), CaCl_2_2.04 × 10^–5^ M (Amresco), NaHCO_3_ 0.1.43 × 10^–4^ M (Sigma-Aldrich),0.05% Tamol^®^ (Dupont, purchased from Northeastern Laboratories), 4% glucose (Amresco), 0.5% N-Z amine (Sigma-Aldrich) was made as previously reported and stored at room temperature (Converse, 1955; Mead et al., 2019). Samples were grown in triplicate. Sterile media controls were treated in the same manner.

*Mice:* Female ICR (CD-1^®^) outbred mice (Envigo) 6–8 weeks of age were used for these studies. Mice were housed according to NIH guidelines for housing and care in a biosafety level 3 animal laboratory. All procedures were approved by the Institutional Animal Care and Use Committee (protocol number 16-009) of Northern Arizona University.

*Pulmonary coccidioidal infection:* ICR mice were anesthetized with ketamine-xylene (80/8 mg/kg) and intranasally inoculated with 1 × 10^6^ of arthroconidia of *C. posadasii* strains C735 or Silveira suspended in 30 μl phosphate-buffered saline (PBS) as described previously (Shubitz et al., 2008). Two mice from each infection group (C735 and Silveira) were sacrificed on day four. The right lobe of the lung was harvested and flash frozen in liquid nitrogen.

### *In vitro* Expression

*Total RNA:* Spherule cell pellets were collected by filtering cultures through 70 μm filters followed by centrifugation at 12,000 × *g* for 15 min at 4°C. Mycelia cultures were centrifuged at 12,000 × *g* for 15 min at 4°C. These pellets were suspended in 1 mL of Trisure (Bioline) and homogenized (Beadbug) with 1.4 mm glass beads for four 30 s cycles at max speed. The tubes were stored on ice between cycles for 5 min. Next, the tubes were inverted at room temperature for 5 min followed by the addition of 1/5 the volume of chloroform. After inverting the mixture for 2 min, the cell debris was pelleted by centrifugation at max speed for 15 min at 4°C. The aqueous phase was collected and placed in 500 μl isopropanol and gently mix at room temperature for 10 min. Nucleic acid was pelleted at max speed for 10 min at 4°C. This pellet was washed with 75% ethanol twice and resuspended in RNAase free water. Total RNA quality was checked using the RNA 6000 nanochip (Agilent).

*RNA sequencing:* mRNA was isolated using the Magnetic mRNA Isolation Kit (New England BioLabs) according to the manufacturers guidelines and prepared for sequencing using NEbNext Ultra Directional Library Prep Kit (New England Biolabs) according to the manufacturer’s guidelines. Stranded, paired-end sequencing was performed using a MiSeq 600-cycle V3 kit (Illumina) with six libraries on a single flow cell.

### *In vitro* Differential Expression Analysis

Raw reads for *C. posadasii* C735 from the previously published Winston data set were obtained from NCBI short read archive using accession number SRA054882. These raw reads and the newly generated mutant reads were aligned to the reference genome, *C. posadasii* C735 SOWgp (GCA_000151335.1) using TopHat2 (Trapnell et al., 2012; Kim et al., 2013). Gene expression quantification and statistical comparison was completed with the Cufflinks package (Trapnell et al., 2012; Kim et al., 2013). These data were visualized using the Cummerbund Package for R (Trapnell et al., 2012; Goff et al., 2019). Analysis was focused on genes that passed the Cufflinks quality filter for sufficient reads that were differentially expressed up or down by twofold change, and after Benjamini-Hochberg False Discovery Rate (FDR) correction were statistically significant (*p* < 0.05) (Benjamini and Hochberg, 1995). Gene product descriptions were obtained from FungiDB (Stajich et al., 2012; Basenko et al., 2018). When *Coccidioides* genes were classified as hypothetical or predicted proteins, ortholog functions were queried using the FungiDB, OrthoMC, and Interpro databases (Li et al., 2003). We used orthologs from *Aspergillus niger* (plant pathogen) and *A. fumigatus* (human pathogen) to investigate significantly enriched gene ontology (GO) terms (Ashburner et al., 2000; Carbon et al., 2009). For the full data set see Supplementary Table 1.

*In silico predictions:* When gene orthologs were *Coccidioides*-specific three prediction programs; NCBI Classifier, EggNOG Mapper, and InterProScan were utilized to predict conserved protein domains. The amino acid sequences were obtained through the FungiDB website. These sequences were submitted to the NCBI, Interproscan and EggNOG Mapper online servers using default parameters. EggNOG Mapper was restricted to the Fungal Kingdom (Jones et al., 2014; Huerta-Cepas et al., 2016, 2017, 2019; Marchler-Bauer et al., 2017; Mitchell et al., 2019). All three predictions are reported in Supplementary Table 6 and Table 3.

### *In vivo* Expression

*Total RNA:* Briefly, for mouse lungs, 30 mg frozen tissue was mixed with 0.5 mm glass beads (Sigma-Aldrich) suspended in 1 ml of Trizol reagent (ThermoFisher Scientific) and homogenized (Beadbug) six times, 30 s per round. Tubes were cooled on ice for 30 s between rounds. Next, 1/5 the volume chloroform was added and mixed well, incubated for 2 min, and homogenized for 15 s. Samples were centrifuged 12,000 × *g* for 15 min at 4°C. The aqueous layer was collected and mixed with 500 μl isopropanol for 10 min and pipetted onto RNeasy spin columns (Qiagen). Columns were centrifuged 10,000 rpm for 1 min, flow through discarded, and washed twice with RPE buffer. Columns were moved to a fresh RNase free 1.5 ml tube and 100 μl RNase free water was added and centrifuged for 1 min and centrifuged to collect nucleic acid. Ten units of RNasin^TM^ Plus RNase Inhibitor (Promega) (1 μ/μl) was added. Extraction method based on (Chung et al., 2014).

*RNA purification:* Purified total RNA quantities of all samples, including *in vitro*, were initially measured using the NanoDrop 1000 Spectrophotometer and verified with the Qubit 2.0 Fluorometric RNA HS assay (Thermo Fisher Scientific). RNA quality was assessed using the Agilent Standard Sensitivity RNA assay with the Fragment Analyzer Automated CE system (Advanced Analytical Technologies). Total RNA extracted from *in vitro Coccidioides* spherules was purified following manufacturer protocol using the RNA Clean & Concentrator-5 kit (Zymo Research) and run through the same quality checks (QC). Purification, additional QC and nCounter gene expression services were performed by the University of Arizona Genetics Core (UAGC, Tucson, AZ, United States).

### NanoString Gene Expression

Total RNA from *in vivo* samples (100–200 ng) and *in vitro* samples (1–200 ng, depending on assay protocol) was used to analyze gene expression changes using nCounter FLEX Analysis System and nSolver 4.0 software (NanoString Technologies Inc.). *Coccidioides* target counts were obtained using a custom panel targeting endogenous *Coccidioides* genes and five internal reference controls, was designed using NanoString Technologies services (NanoString Technologies) and performed by UAGC.

Based on RNA integrity, 200 ng purified total RNA was initially hybridized to reporter and capture barcoded probe sets using the NanoString assay XT protocol at 65°C for 19 h. Samples were processed on an nCounter FLEX Analysis system (NanoString Technologies Inc.). Purification and binding of the hybridized probes to the optical cartridge was performed on the nCounter Prep Station using high sensitivity settings, and the cartridge was scanned on the nCounter Digital Analyzer using Field of View setting (FOV) of 555. Raw counts from each gene were imported into the nSolver Analysis Software and normalized against background and housekeeping genes. Data were normalized using internal positive and negative controls and selected housekeeping genes (CIMG_01599, CIMG_10083, and CIMG_12902) and overall assay performance was assessed through evaluation of built-in positive controls.

After initial results from the *Coccidioides* panel, the *Coccidioides* panel sample set was repeated using the NanoString Low RNA Input protocol (NanoString Technologies Inc.) for cDNA synthesis and multiplexed targeted enrichment (MTE) of *Coccidioides* targets. Depending on strain and previous results, 10–100 ng total inoculated mouse RNA or 1 ng *in vitro Coccidioides* RNA was cDNA-converted and amplified. This was done using custom-designed cocci-specific MTE oligonucleotide pool [Integrated DNA Technologies (IDT)] for 8 cycles in an MJ Research PTC-225 Peltier Thermal Cycler (Bio-Rad) following NanoString Low RNA Input protocol cycling conditions.

### *In vitro* Volatile Metabolome

*Sample Collection:* Spherules of C735 and the attenuated mutant strain and sterile media controls were pelleted at 12,000 × *g* at 4°C for 10 min, the supernatant was placed in a 0.22 μm spin filter and centrifuged at 4,000 rpm for 4 min. The filtrate was stored at −80°C until volatile metabolomics analysis. The samples were allowed to thaw at 4°C overnight, and then 2 ml were transferred and sealed into sterilized 10 ml GC headspace vials with PTFE/silicone septum screw caps. Three biological replicates each of wild type, mutant, and Converse media blanks were prepared. All samples were stored for up to 10 days at 4°C until analyzed.

*Volatile metabolite sampling*: Measurements were performed using a Gerstel Multipurpose Sampler directed by Maestro software. Two-dimensional gas chromatography-time-of-flight mass spectrometry (GC × GC–TOFMS) was performed using a LECO Pegasus 4D and Agilent 7890 GC with chromatographic, mass spectrometric, and peak detection parameters provided in Supplementary Table 10. An external alkane standards mixture (C_8_ – C_20_; Sigma-Aldrich), was sampled multiple times for use in determining retention indices (RI). The injection, chromatographic, and mass spectrometric methods for analyzing the alkane standards were the same as for the samples. All conditions and parameters are listed in Supplementary Table 10.

### Volatile Compound Analysis

Compound Processing: Data collection, processing, and alignment were performed using ChromaTOF software version 4.71 with the Statistical Compare package (Leco Corp.), using parameters listed in Supplementary Table 10. Peaks were identified by forward and reverse searches of the NIST 2011 mass spectral library. Peaks were assigned a putative identification based on mass spectral similarity and retention index (RI) data, and the confidence of those identifications are indicated by assigning levels 1–4 (with 2 being the highest in this study) (Sumner et al., 2007). Peaks with a level 2 identification were named, and were identified based on ≥800 mass spectral match by a forward and reverse search of the NIST 2011 mass spectral library and RI that are consistent with the midpolar Rxi-624Sil stationary phase, as previously described (Bean et al., 2016), but using an RI range of 0–43%, which was empirically determined by comparing the Rxi-624Sil RIs for Grob mix standards to published polar and non-polar values. Level 2 and 3 compounds were assigned to chemical functional groups based upon characteristic mass spectral fragmentation patterns and second dimension retention times. Level 4 compounds have mass spectral matches <600 and are reported as unknowns.

### Volatile Statistical Analysis

Before statistical analyses, compounds eluting prior to 358 s (acetone retention time) and siloxanes (i.e., chromatographic artifacts) were removed from the peak table. Peaks that were present in only one of the three biological replicates were imputed to zero for all three replicates. Peaks that were present in two out of three biological replicates were imputed to half of the minimum value of the two other biological replicates. The relative abundance of compounds across chromatograms was normalized using probabilistic quotient normalization (PQN) (Dieterle et al., 2006) and log_10_ transformed in R version 3.4.3. Intraclass correlation coefficients (ICCs) were calculated, using R ICC package version 2.3.0, and peaks with an ICC < 0.9 were not further processed. Analytes were retained for further analysis if they were present in one strain but absent in media and the other strain (uniquely produced) or if they were present in media and one strain but absent in the other strain (uniquely consumed). Wilcoxon Rank Sum test with Benjamini and Hotchberg FDR correction was performed with α = 0.05, using R stats package version 3.5.3.

### Transmission Electron Microscopy

After 96 h of growth *C. posadasii* strain C735 and Δ*cts2*/Δ*ard1*/Δ*cts3* spherules were collected by filtering cultures through 70 μm filters (Corning) and centrifuged at 12,000 × *g* for 15 min at 4°C. Pellets were re-suspended in 1% formalin for 72 h. Following fixation, the cells were centrifuged at 10,000 × *g* for 5 min and washed in PBS [Electron Microscopy Sciences, (EMS)] for 10 min, three times. The pellet was then fixed in 2.5% glutaraldehyde (EMS) in PBS overnight at 4°C. Cells were pelleted and washed in PBS for 10 min, three times. Next, cells were suspended in 2% sodium alginate, dropped into cold calcium chloride, and then chilled at 4°C for 1 h to form concentrated beads. The chilled cells were washed in PBS for 10 min, twice. Osmium tetroxide, 1% in H_2_O (EMS) was added to the sodium alginate beads and left for 30 min at room temperature. The beads were washed in water for 10 min, three times. These beads were subjected to a dehydration series; 50% ethanol for 10 min, 70% ethanol overnight at 4°C, in 95% ethanol for 10 min and 100% ethanol for 10 min, three times. The beads were then infiltrated with a 50:50 mixture of 100% ethanol and Spurrs’ resin (EMS) for 3 h on a rotator followed by a 3:1 ratio of Spurrs’ resin to 100% ethanol overnight. The beads were subjected to several changes of 100% Spurrs’ resin over a 2 day period on a rotatator before being embedded in BEEM capsules and cured at 70°C for 3 days in an oven. The resulting resin blocks of both wild-type and the attenuated mutant spherules were then trimmed using a microtome and razor blade. Thick sections were cut and stained with toluidine blue to confirm spherule location before 50 nm thin sections were cut and placed on 200 mesh copper grids. Grids were left to dry and then post-stained with Uranyless (EMS) for 30 min and lead citrate (EMS) for 12 min and left to dry. Sections on grids were then analyzed with a JEOL 1200 EX-II TEM at 60 kV. Images were taken from all five blocks and from deep within the beads as verified with thick sections in order to ensure accurate representation of both wild type and the attenuated mutant.

## Data Availability Statement

The datasets generated for this study have been deposited at NCBI using project accession number PRJNA608815, https://www.ncbi.nlm.nih.gov/sra/PRJNA608815.

## Ethics Statement

The animal study was reviewed and approved by Northern Arizona University Institutional Animal Care and Use Committee.

## Author Contributions

HM analyzed data and wrote manuscript, performed differential expression data analysis. HM performed the *in vitro* experiments with assistance from KM. CR assisted HM with raw read analysis. MV and HM performed *in vivo* RNA extractions and MV performed NanoString Analysis. EH performed volatile collection and analysis with supervision and support from HB. KL and AF processed samples and captured TEM images. MV, CR, HB, BB, and JS provided manuscript edits. BB funded, supervised and advised throughout the study. All authors read and approved of the manuscript.

## Conflict of Interest

The authors declare that the research was conducted in the absence of any commercial or financial relationships that could be construed as a potential conflict of interest.
