# Supplementary material for: Defining Critical Genes During Spherule Remodeling and Endospore Development in the Fungal Pathogen, Coccidioides posadasii
Source: Front Genet. 2020 May 15;11:483. doi: 10.3389/fgene.2020.00483 (PMC7243461; doi:10.3389/fgene.2020.00483)
Supplement: Supplementary file 1 [file Image_1.PDF]

frontiers

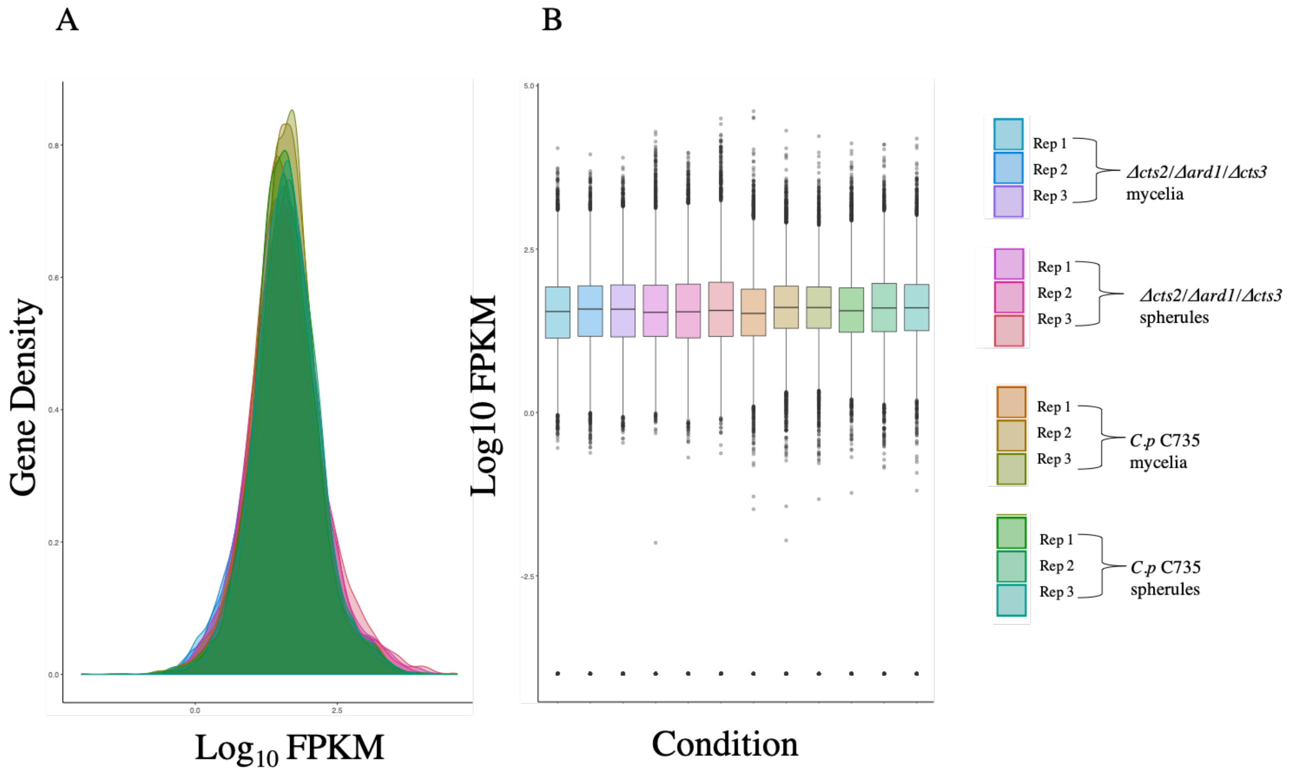

Supplemental Figure 1. Visualization of transcript density for each sample and boxplots for all replicates. The figure legend applies to both plots. (A) There is a similar distribution of FPKM scores across biological replicates. (B) Log_{10} FPKM values for individual replicates. Biological replicates show a high degree of similarity for each sample.
