# Supplementary material for: Defining Critical Genes During Spherule Remodeling and Endospore Development in the Fungal Pathogen, Coccidioides posadasii
Source: Front Genet. 2020 May 15;11:483. doi: 10.3389/fgene.2020.00483 (PMC7243461; doi:10.3389/fgene.2020.00483)
Supplement: Supplementary file 2 [file Image_2.PDF]

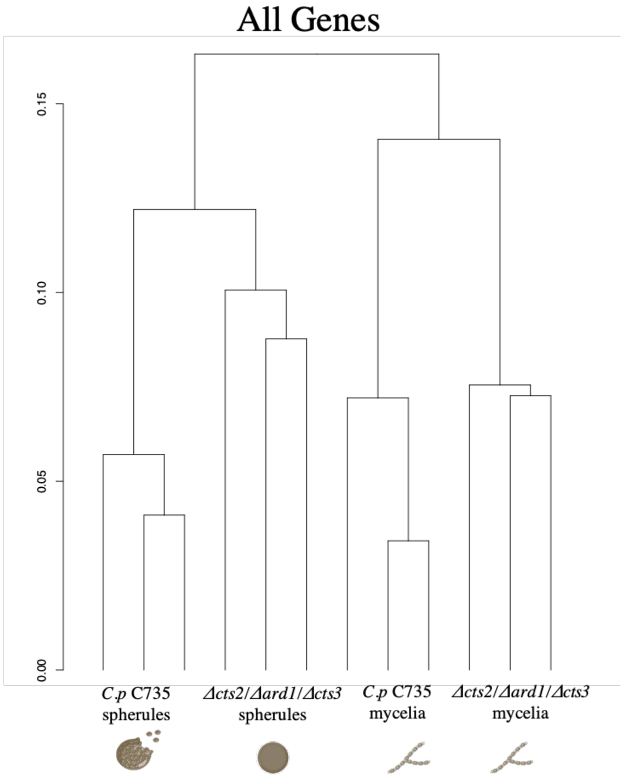

Supplemental Figure 2. Dendrogram of individual biological replicates for *C. posadasii* C735 wild-type and Δ*cts2*/Δ*ard1*/Δ*cts3* mutant. A Jensen-Shannon divergence of FPKM values which show that genes cluster by lifecycle stage rather than strain. The Δ*cts2*/Δ*ard1*/Δ*cts3* strain is derived from C735.
