# Supplementary material for: Defining Critical Genes During Spherule Remodeling and Endospore Development in the Fungal Pathogen, Coccidioides posadasii
Source: Front Genet. 2020 May 15;11:483. doi: 10.3389/fgene.2020.00483 (PMC7243461; doi:10.3389/fgene.2020.00483)
Supplement: Supplementary file 5 [file Table_3.DOCX]

Supplemental Table 3. Gene ontology for enriched biological processes for the 19 improperly down-regulated genes in mutant spherules. Gene ontology based on *A. niger* (white) and *A. fumigatus* (grey) suggests the mutant strain displays improper regulation of genes related to metal homeostasis. Specifically, siderophore transcription factors, iron, and copper acquisition.
