# Supplementary material for: Defining Critical Genes During Spherule Remodeling and Endospore Development in the Fungal Pathogen, Coccidioides posadasii
Source: Front Genet. 2020 May 15;11:483. doi: 10.3389/fgene.2020.00483 (PMC7243461; doi:10.3389/fgene.2020.00483)
Supplement: Supplementary file 7 [file Table_5.docx]

Supplemental Table 5. Complete gene list, predicted function and ortholog groups for 222 genes up-regulated 2-fold or more in *C. posadasii* strain C735 spherules compared to mycelia. *Coccidioides* specific genes are highlighted in grey.

| Gene ID | Product Description | Ortholog count | Ortholog Group |
| --- | --- | --- | --- |
| CPC735_012730 | conserved hypothetical protein | 8 | OG5_187736 |
| CPC735_037430 | hypothetical protein | 0 | ORTH_52755.tmp |
| CPC735_039170 | hypothetical protein | 2 | OG5_cpos\|CPAG_00602 |
| CPC735_048280 | hypothetical protein | 4 | OG5_223675 |
| CPC735_054380 | hypothetical protein | 2 | OG5_cpos\|CPAG_01987 |
| CPC735_055710 | hypothetical protein | 1 | OG5_cpos\|CPAG_02162 |
| CPC735_067770 | hypothetical protein | 4 | OG5_223479 |
| CPC735_040660 | predicted protein | 3 | OG5_cimm\|CIMG_02439T0 |
| CPC735_062130 | srpk, putative | 2 | OG5_cpos\|CPAG_05053 |
| CPC735_006580 | hypothetical protein | 12 | OG5_210714 |
| CPC735_020310 | hypothetical protein | 1 | OG5_uree\|UREG_02272 |
| CPC735_033840 | hypothetical protein | 88 | OG5_131078 |
| CPC735_055730 | hypothetical protein | 199 | OG5_129758 |
| CPC735_058940 | hypothetical protein | 251 | OG5_129350 |
| CPC735_070310 | hypothetical protein | 147 | OG5_129256 |
| CPC735_049760 | 4-hydroxyphenylpyruvate dioxygenase , putative | 183 | OG5_129448 |
| CPC735_026260 | 5-aminolevulinate synthase, mitochondrial precursor , putative | 309 | OG5_126783 |
| CPC735_061990 | ABC1 family protein | 147 | OG5_128411 |
| CPC735_005490 | acetate kinase family protein | 119 | OG5_130287 |
| CPC735_063030 | acetyl-CoA hydrolase, putative | 103 | OG5_131950 |
| CPC735_047580 | aconitate hydratase, mitochondrial precursor, putative | 355 | OG5_126691 |
| CPC735_021820 | Acyl-CoA desaturase, putative | 266 | OG5_126939 |
| CPC735_059740 | agmatinase, putative | 319 | OG5_127636 |
| CPC735_068280 | alcohol dehydrogenase, putative | 126 | OG5_149662 |
| CPC735_007960 | allantoicase, putative | 142 | OG5_130881 |
| CPC735_072410 | Alpha amylase, catalytic domain containing protein | 277 | OG5_130769 |
| CPC735_050450 | alpha/beta hydrolase fold domain containing protein | 166 | OG5_141437 |
| CPC735_053100 | Alternative oxidase, mitochondrial precursor, putative | 218 | OG5_130283 |
| CPC735_030160 | aminotransferase, classes I and II family protein | 67 | OG5_163698 |
| CPC735_047350 | aminotransferase, classes I and II family protein | 171 | OG5_128841 |
| CPC735_028320 | ankyrin repeat containing protein | 1334 | OG5_126538 |
| CPC735_012290 | aquaporin-7, putative | 623 | OG5_126615 |
| CPC735_071000 | aspartyl-tRNA synthetase, putative | 290 | OG5_127227 |
| CPC735_054090 | ATPase, AAA family protein | 5 | OG5_211131 |
| CPC735_010590 | Auxin Efflux Carrier family protein | 148 | OG5_132929 |
| CPC735_005830 | BAG domain containing protein | 47 | OG5_164000 |
| CPC735_026130 | BAR domain containing protein | 118 | OG5_129271 |
| CPC735_049390 | C2 domain containing protein | 115 | OG5_146798 |
| CPC735_018430 | C2H2 type zinc finger containing protein | 65 | OG5_163470 |
| CPC735_063550 | carboxyphosphonoenolpyruvate mutase, putative | 77 | OG5_146820 |
| CPC735_012670 | Catalase A, putative | 476 | OG5_127182 |
| CPC735_058480 | chitinase | 317 | OG5_130746 |
| CPC735_010220 | CybS family protein | 169 | OG5_129488 |
| CPC735_058920 | Cyclin, N-terminal domain containing protein | 165 | OG5_129826 |
| CPC735_068200 | Cytidine and deoxycytidylate deaminase zinc-binding domain containing protein | 94 | OG5_149799 |
| CPC735_068100 | cytochrome c, putative | 154 | OG5_127365 |
| CPC735_031520 | Cytochrome P450 family protein | 57 | OG5_159373 |
| CPC735_048650 | D-isomer specific 2-hydroxyacid dehydrogenase family protein | 373 | OG5_126787 |
| CPC735_048940 | D-isomer specific 2-hydroxyacid dehydrogenase, NAD binding domain containing protein | 93 | OG5_140208 |
| CPC735_062250 | Deuterolysin metalloprotease family protein | 4 | OG5_223492 |
| CPC735_049950 | di/tri peptide transporter, putative | 512 | OG5_126717 |
| CPC735_040920 | dimeric dihydrodiol dehydrogenase, putative | 32 | OG5_146501 |
| CPC735_009690 | Dioxygenase family protein | 124 | OG5_139326 |
| CPC735_021540 | Dynamin family protein | 56 | OG5_172020 |
| CPC735_035370 | FAD binding domain containing protein | 340 | OG5_133483 |
| CPC735_024720 | Fasciclin domain containing protein | 87 | OG5_149337 |
| CPC735_023590 | fatty acid oxygenase, putative | 241 | OG5_129768 |
| CPC735_009780 | ferric reductase transmembrane component, putative | 91 | OG5_152682 |
| CPC735_035740 | Flavin-binding monooxygenase-like family protein | 83 | OG5_146853 |
| CPC735_071880 | Flavin-binding monooxygenase-like family protein | 248 | OG5_137003 |
| CPC735_063010 | flavocytochrome c family protein | 221 | OG5_128620 |
| CPC735_013760 | Fungal Zn binuclear cluster domain containing protein | 29 | OG5_175847 |
| CPC735_062170 | Fungal Zn binuclear cluster domain containing protein | 11 | OG5_223927 |
| CPC735_073370 | Glucosamine-6-phosphate isomerase , putative | 177 | OG5_128189 |
| CPC735_011710 | glutamate decarboxylase, putative | 253 | OG5_129592 |
| CPC735_057270 | glutaryl-CoA dehydrogenase, putative | 151 | OG5_129299 |
| CPC735_055250 | Glutathione S-transferase, putative | 465 | OG5_126942 |
| CPC735_040340 | glycosyl hydrolase, family 43 protein | 167 | OG5_137411 |
| CPC735_073380 | glycosyl hydrolase, putative | 176 | OG5_130549 |
| CPC735_028170 | glycosyl transferase, group 1 family protein | 158 | OG5_138640 |
| CPC735_046840 | HAD-superfamily subfamily IIA hydrolase, TIGR01456, CECR5 containing protein | 342 | OG5_127847 |
| CPC735_070990 | Hemerythrin family protein | 156 | OG5_139259 |
| CPC735_049570 | HMG box domain containing protein | 105 | OG5_146233 |
| CPC735_047390 | Hsp20/alpha crystallin family protein | 184 | OG5_126935 |
| CPC735_000820 | hypothetical protein | 105 | OG5_136572 |
| CPC735_000990 | hypothetical protein | 84 | OG5_152860 |
| CPC735_001320 | hypothetical protein | 88 | OG5_138628 |
| CPC735_001550 | hypothetical protein | 88 | OG5_169375 |
| CPC735_004470 | hypothetical protein | 69 | OG5_159101 |
| CPC735_004620 | hypothetical protein | 70 | OG5_140219 |
| CPC735_006560 | hypothetical protein | 119 | OG5_136130 |
| CPC735_007950 | hypothetical protein | 85 | OG5_155382 |
| CPC735_009400 | hypothetical protein | 36 | OG5_223062 |
| CPC735_009490 | hypothetical protein | 98 | OG5_140223 |
| CPC735_009620 | hypothetical protein | 61 | OG5_168977 |
| CPC735_010250 | hypothetical protein | 75 | OG5_163404 |
| CPC735_010730 | hypothetical protein | 96 | OG5_155947 |
| CPC735_011120 | hypothetical protein | 85 | OG5_152638 |
| CPC735_011730 | hypothetical protein | 5 | OG5_223608 |
| CPC735_011760 | hypothetical protein | 76 | OG5_152855 |
| CPC735_011950 | hypothetical protein | 53 | OG5_164048 |
| CPC735_012560 | hypothetical protein | 50 | OG5_155929 |
| CPC735_012570 | hypothetical protein | 50 | OG5_169325 |
| CPC735_013640 | hypothetical protein | 98 | OG5_159125 |
| CPC735_014840 | hypothetical protein | 119 | OG5_144519 |
| CPC735_014890 | hypothetical protein | 194 | OG5_140625 |
| CPC735_017030 | hypothetical protein | 52 | OG5_155905 |
| CPC735_017210 | hypothetical protein | 9 | OG5_223985 |
| CPC735_017860 | hypothetical protein | 7 | OG5_223555 |
| CPC735_018390 | hypothetical protein | 240 | OG5_137954 |
| CPC735_018490 | hypothetical protein | 77 | OG5_159293 |
| CPC735_018590 | hypothetical protein | 124 | OG5_146762 |
| CPC735_018870 | hypothetical protein | 136 | OG5_144599 |
| CPC735_022170 | hypothetical protein | 5 | OG5_223463 |
| CPC735_024050 | hypothetical protein | 69 | OG5_159156 |
| CPC735_024190 | hypothetical protein | 114 | OG5_137935 |
| CPC735_024320 | hypothetical protein | 73 | OG5_133139 |
| CPC735_025020 | hypothetical protein | 191 | OG5_132830 |
| CPC735_026040 | hypothetical protein | 13 | OG5_223771 |
| CPC735_027750 | hypothetical protein | 5 | OG5_223295 |
| CPC735_028830 | hypothetical protein | 149 | OG5_134272 |
| CPC735_029960 | hypothetical protein | 70 | OG5_159478 |
| CPC735_029970 | hypothetical protein | 148 | OG5_137982 |
| CPC735_031960 | hypothetical protein | 119 | OG5_135783 |
| CPC735_032610 | hypothetical protein | 97 | OG5_135112 |
| CPC735_034270 | hypothetical protein | 20 | OG5_169838 |
| CPC735_034870 | hypothetical protein | 9 | OG5_187473 |
| CPC735_035630 | hypothetical protein | 51 | OG5_187971 |
| CPC735_035730 | hypothetical protein | 100 | OG5_152877 |
| CPC735_035800 | hypothetical protein | 50 | OG5_163724 |
| CPC735_036330 | hypothetical protein | 82 | OG5_152281 |
| CPC735_036790 | hypothetical protein | 162 | OG5_134531 |
| CPC735_037080 | hypothetical protein | 38 | OG5_180711 |
| CPC735_041990 | hypothetical protein | 83 | OG5_149935 |
| CPC735_042180 | hypothetical protein | 94 | OG5_155963 |
| CPC735_042220 | hypothetical protein | 72 | OG5_159616 |
| CPC735_042590 | hypothetical protein | 80 | OG5_141396 |
| CPC735_043920 | hypothetical protein | 97 | OG5_139407 |
| CPC735_045800 | hypothetical protein | 13 | OG5_cpos\|CPAG_00855 |
| CPC735_045940 | hypothetical protein | 93 | OG5_139364 |
| CPC735_046700 | hypothetical protein | 167 | OG5_136103 |
| CPC735_047230 | hypothetical protein | 25 | OG5_176313 |
| CPC735_047550 | hypothetical protein | 73 | OG5_158914 |
| CPC735_048620 | hypothetical protein | 92 | OG5_155839 |
| CPC735_049490 | hypothetical protein | 93 | OG5_176037 |
| CPC735_050810 | hypothetical protein | 95 | OG5_152750 |
| CPC735_053240 | hypothetical protein | 223 | OG5_134262 |
| CPC735_053450 | hypothetical protein | 63 | OG5_163567 |
| CPC735_055630 | hypothetical protein | 90 | OG5_155895 |
| CPC735_055670 | hypothetical protein | 80 | OG5_163648 |
| CPC735_057240 | hypothetical protein | 125 | OG5_159308 |
| CPC735_059020 | hypothetical protein | 100 | OG5_159286 |
| CPC735_059050 | hypothetical protein | 34 | OG5_169826 |
| CPC735_060300 | hypothetical protein | 7 | OG5_223638 |
| CPC735_060560 | hypothetical protein | 274 | OG5_131769 |
| CPC735_063230 | hypothetical protein | 5 | OG5_223504 |
| CPC735_066080 | hypothetical protein | 57 | OG5_159632 |
| CPC735_066570 | hypothetical protein | 154 | OG5_136546 |
| CPC735_066660 | hypothetical protein | 15 | OG5_223241 |
| CPC735_066900 | hypothetical protein | 144 | OG5_146869 |
| CPC735_067310 | hypothetical protein | 87 | OG5_163422 |
| CPC735_067460 | hypothetical protein | 29 | OG5_169787 |
| CPC735_067760 | hypothetical protein | 12 | OG5_176276 |
| CPC735_068000 | hypothetical protein | 59 | OG5_180757 |
| CPC735_068350 | hypothetical protein | 194 | OG5_140625 |
| CPC735_068750 | hypothetical protein | 5 | OG5_223741 |
| CPC735_068820 | hypothetical protein | 6 | OG5_223199 |
| CPC735_070700 | hypothetical protein | 102 | OG5_139306 |
| CPC735_070920 | hypothetical protein | 114 | OG5_133251 |
| CPC735_072050 | hypothetical protein | 97 | OG5_152470 |
| CPC735_073260 | hypothetical protein | 102 | OG5_147021 |
| CPC735_031570 | ImpB/MucB/SamB family protein | 153 | OG5_128521 |
| CPC735_049700 | Iron-sulfur cluster assembly accessory family protein | 150 | OG5_127222 |
| CPC735_033010 | Lanthionine synthetase C-like protein | 99 | OG5_128365 |
| CPC735_002410 | Longevity-assurance family protein | 228 | OG5_126838 |
| CPC735_071520 | magnesium-translocating P-type ATPase family protein | 417 | OG5_127253 |
| CPC735_010130 | major facilitator superfamily protein | 91 | OG5_149926 |
| CPC735_014400 | Major Facilitator Superfamily protein | 114 | OG5_155540 |
| CPC735_014480 | Major Facilitator Superfamily protein | 126 | OG5_149776 |
| CPC735_032680 | Major Facilitator Superfamily protein | 369 | OG5_134002 |
| CPC735_049550 | Major Facilitator Superfamily protein | 317 | OG5_132562 |
| CPC735_053500 | Major Facilitator Superfamily protein | 177 | OG5_136118 |
| CPC735_063110 | Major Facilitator Superfamily protein | 1167 | OG5_126664 |
| CPC735_012310 | Malate synthase, glyoxysomal, putative | 189 | OG5_130009 |
| CPC735_052400 | mariner-Tc1 transposon family protein | 270 | OG5_127357 |
| CPC735_051410 | methionine-R-sulfoxide reductase family protein | 162 | OG5_127019 |
| CPC735_002470 | Mitochondrial carrier protein | 138 | OG5_140273 |
| CPC735_048820 | Mn-superoxide dismutase, putative | 287 | OG5_126676 |
| CPC735_057020 | multidrug resistance ABC transporter, putative | 2853 | OG5_126574 |
| CPC735_071320 | myb family transcription factor | 487 | OG5_127030 |
| CPC735_003840 | NADH-ubiquinone oxidoreductase 23 kDa subunit, mitochondrial precursor, putative | 144 | OG5_128019 |
| CPC735_063880 | NmrA-like family protein | 276 | OG5_131898 |
| CPC735_067410 | non-classical export protein, putative | 102 | OG5_137413 |
| CPC735_071370 | O-methyltransferase, putative | 62 | OG5_149870 |
| CPC735_020560 | Oxidoreductase molybdopterin binding domain containing protein | 487 | OG5_126955 |
| CPC735_029260 | Oxidoreductase molybdopterin binding domain containing protein | 186 | OG5_129905 |
| CPC735_030560 | oxidoreductase, short chain dehydrogenase/reductase family protein | 72 | OG5_163465 |
| CPC735_053080 | oxidoreductase, short chain dehydrogenase/reductase family protein | 196 | OG5_128170 |
| CPC735_023020 | oxidoreductase,short chain dehydrogenase, putative | 151 | OG5_135785 |
| CPC735_034980 | Patatin-like phospholipase family protein | 109 | OG5_135392 |
| CPC735_026690 | peroxisomal copper amine oxidase, putative | 428 | OG5_127171 |
| CPC735_047130 | PH domain containing protein | 190 | OG5_136164 |
| CPC735_018090 | PHD-finger motif containing protein | 118 | OG5_134802 |
| CPC735_001330 | phosphatase regulatory subunit family protein | 7 | OG5_133458 |
| CPC735_065470 | phosphatase regulatory subunit family protein | 226 | OG5_134990 |
| CPC735_039500 | Phosphatidylethanolamine-binding protein | 236 | OG5_127642 |
| CPC735_005500 | phosphoketolase, putative | 170 | OG5_136541 |
| CPC735_038600 | pirin domain containing protein | 270 | OG5_128233 |
| CPC735_071300 | polyketide synthase, putative | 197 | OG5_134266 |
| CPC735_029370 | potassium uptake protein, Trk family protein | 238 | OG5_131516 |
| CPC735_003620 | PQ loop repeat family protein | 263 | OG5_128454 |
| CPC735_041080 | predicted protein | 32 | OG5_163427 |
| CPC735_035890 | Protein kinase domain containing protein | 6 | OG5_160953 |
| CPC735_002770 | Putative tyrosine phosphatase family protein | 163 | OG5_131528 |
| CPC735_055750 | pyridoxamine 5'-phosphate oxidase, putative | 144 | OG5_129127 |
| CPC735_005250 | pyruvate decarboxylase, putative | 266 | OG5_129158 |
| CPC735_028620 | Ras family protein | 163 | OG5_129742 |
| CPC735_070300 | RmlD substrate binding domain containing protein | 130 | OG5_134187 |
| CPC735_073220 | RNA recognition motif containing protein | 141 | OG5_132162 |
| CPC735_058150 | RNA-directed RNA polymerase, putative | 247 | OG5_130286 |
| CPC735_014970 | Serine palmitoyltransferase 2 , putative | 226 | OG5_127265 |
| CPC735_067680 | SNARE domain containing protein | 156 | OG5_127926 |
| CPC735_020780 | sphingolipid long chain base-responsive protein PIL1, putative | 135 | OG5_134534 |
| CPC735_018890 | sterigmatocystin 8-O-methyltransferase precursor, putative | 143 | OG5_152897 |
| CPC735_051240 | steroid monooxygenase, putative | 28 | OG5_cimm\|CIMG_01276T0 |
| CPC735_051790 | sterol desaturase, putative | 141 | OG5_133261 |
| CPC735_047380 | subtilisin-like protease, putative | 5 | OG5_215783 |
| CPC735_033080 | succinyl-CoA synthetase beta chain, putative | 96 | OG5_159139 |
| CPC735_051270 | sulfate permease, putative | 512 | OG5_126650 |
| CPC735_005240 | THUMP domain containing protein | 165 | OG5_129535 |
| CPC735_038590 | Tripeptidyl-peptidase I precursor, putative | 358 | OG5_130135 |
| CPC735_056200 | zinc knuckle containing protein | 81 | OG5_159448 |
