# Supplementary material for: Defining Critical Genes During Spherule Remodeling and Endospore Development in the Fungal Pathogen, Coccidioides posadasii
Source: Front Genet. 2020 May 15;11:483. doi: 10.3389/fgene.2020.00483 (PMC7243461; doi:10.3389/fgene.2020.00483)
Supplement: Supplementary file 8 [file Table_6.docx]

Supplemental Table 6. Eight *Coccidioides* specific hypothetical proteins that are significantly up-regulated 2-fold or more in the wild-type lifecycle (mycelia vs spherules) but not in the mutant. Results of *in silico* predictions based on amino acid sequence are listed. These proteins could be specific to endospore formation, release or disease establishment

| *Coccidioides* Specific Gene Description | | | Conserved Domains | | Fold Change  Mycelia to Spherule | |
| --- | --- | --- | --- | --- | --- | --- |
| Gene ID | **Product Description** | **NCBI** | **InterproScan** | **EggNOG Mapper** | **Δ*cts2*/Δ*ard1*/Δ*cts3*** | ***C. posadasii* C735** |
| CPC735_012730 | conserved hypothetical protein | Pyoverdine/dityrosinebiosynthes is protein. pfam 05141, E-value: 1.39e-151 | Pyoverdine/dityrosine biosynthesis protein IPR007817 | Pyoverdine/ dityrosine E-value: 5.1e-231 | 1.08367 | 2.40394 |
| CPC735_037430 | hypothetical protein | no hits | No predictions | no hits | 1.37795 | 2.14105 |
| CPC735_039170 | hypothetical protein | Protein kinase like E-value: 8.12e-05 | Protein kinase like/IPR011009 | Phosphotransferase enzyme family protein  E-value:1e-93 | 1.08168 | 2.01994 |
| CPC735_040660 | predicted protein | no hits | Protein kinase like  IPR011009 | no hits | 1.71838 | 2.60077 |
| CPC735_048280 | hypothetical protein | no hits | No predictions | HNH endonuclease  E-value: 3.5e-31 | 3.60023 (q-value 0.0622865) | 4.814 |
| CPC735_054380 | hypothetical protein | no hits | No predictions | HNH endonuclease. E-value: 2.7e-07 | 1.47176 | 2.36553 |
| CPC735_055710 | hypothetical protein | no hits | No predictions | no hits | -0.302669 | 2.21015 |
| CPC735_067770 | hypothetical protein | no hits | No predictions | no hits | 1.81292 | 2.55258 |
