# Supplementary material for: Defining Critical Genes During Spherule Remodeling and Endospore Development in the Fungal Pathogen, Coccidioides posadasii
Source: Front Genet. 2020 May 15;11:483. doi: 10.3389/fgene.2020.00483 (PMC7243461; doi:10.3389/fgene.2020.00483)
Supplement: Supplementary file 9 [file Table_7.docx]

Supplemental Table 7. GO terms for the 151 transcripts significantly (FDR, p < 0.001) up-regulated in wild-type spherules compared to mutant spherules indicate enrichment of biological process specific to proton transport across the membrane, response to oxidative stress and amino acid/carboxylic acid transport.
