# Supplementary material for: Defining Critical Genes During Spherule Remodeling and Endospore Development in the Fungal Pathogen, Coccidioides posadasii
Source: Front Genet. 2020 May 15;11:483. doi: 10.3389/fgene.2020.00483 (PMC7243461; doi:10.3389/fgene.2020.00483)
Supplement: Supplementary file 11 [file Table_9.docx]

Supplemental Table 9. Measurements from TEM images of cell wall thickness and nucleus diameter between wild-type and mutant spherules. The mutant strain nuclei are substantially larger than wild-type. Images are provided in Figure 10.

| Panel | Spherule Type | Mean Cell Wall Thickness (µm) | Mean Nucleus Diameter  (µm) |
| --- | --- | --- | --- |
| A | Wild type | 0.283 | 0.439 |
| B | Wild type | 0.277 | 0.535 |
| C | Wild type | 0.209 | N/A |
| D | Mutant | 0.287 | 1.452 |
| E | Mutant | 0.335 | 1.372 |
| F | Mutant | 0.331 | 2.039 |
